# Effects of environmental conditions on COVID-19 morbidity as an example of multicausality: a multi-city case study in Italy

**DOI:** 10.3389/fpubh.2023.1222389

**Published:** 2023-10-25

**Authors:** Andrea Murari, Michela Gelfusa, Teddy Craciunescu, Claudio Gelfusa, Pasquale Gaudio, Gianluigi Bovesecchi, Riccardo Rossi

**Affiliations:** ^1^Consorzio RFX (CNR, ENEA, INFN, Università di Padova, Acciaierie Venete SpA), Padua, Italy; ^2^Istituto per la Scienza e la Tecnologia dei Plasmi, CNR, Padua, Italy; ^3^Department of Industrial Engineering, University of Rome “Tor Vergata”, Rome, Italy; ^4^National Institute for Laser, Plasma and Radiation Physics, Măgurele, Romania; ^5^Department of Enterprise Engineering, University of Rome “Tor Vergata”, Rome, Italy

**Keywords:** COVID-19, air quality, pollutants, particulate, wind, public policies, traffic

## Abstract

The coronavirus disease 2019 (COVID-19), caused by the severe acute respiratory syndrome coronavirus 2 (SARS-CoV-2), broke out in December 2019 in Wuhan city, in the Hubei province of China. Since then, it has spread practically all over the world, disrupting many human activities. In temperate climates overwhelming evidence indicates that its incidence increases significantly during the cold season. Italy was one of the first nations, in which COVID-19 reached epidemic proportions, already at the beginning of 2020. There is therefore enough data to perform a systematic investigation of the correlation between the spread of the virus and the environmental conditions. The objective of this study is the investigation of the relationship between the virus diffusion and the weather, including temperature, wind, humidity and air quality, before the rollout of any vaccine and including rapid variation of the pollutants (not only their long term effects as reported in the literature). Regarding them methodology, given the complexity of the problem and the sparse data, robust statistical tools based on ranking (Spearman and Kendall correlation coefficients) and innovative dynamical system analysis techniques (recurrence plots) have been deployed to disentangle the different influences. In terms of results, the evidence indicates that, even if temperature plays a fundamental role, the morbidity of COVID-19 depends also on other factors. At the aggregate level of major cities, air pollution and the environmental quantities affecting it, particularly the wind intensity, have no negligible effect. This evidence should motivate a rethinking of the public policies related to the containment of this type of airborne infectious diseases, particularly information gathering and traffic management.

## 1. Introduction: COVID-19 and the weather fast variations

In the last years, it has become clear that, in the present interconnected world, the spread of infectious diseases is one of the major threats to human health and economic activities (1–3). The most evident last case is the outbreak of the severe acute respiratory syndrome coronavirus 2 SARS-CoV-2, which first appeared on a small scale in November 2019 with the first large cluster developing in Wuhan city, in the Hubei province of China in December 2019, before spreading rapidly all over the planet (4, 5). The transmission of coronavirus can occur in different ways. Some of the most important infection routes are interactions with infectious individuals, virus-carrying aerosols, contact with infected surfaces and super spread events due to congested living and traveling conditions (6–16).

Even if the basic forms of direct and indirect transmission are well known, their relative importance is yet not fully understood. Indeed, SARS-CoV-2 has been detected in almost all countries on earth but the transmission dynamics, virulence and mortality have shown substantial heterogeneity across nations, regions, and even neighborhoods (17–19). These spatio-temporal variations certainly depend on several factors, including no pharmaceutical interventions, human behavior, public policies and structural determinants of health (20–26). The influence of the weather and air quality is one of the aspects, which is less understood and certainly requires further investigation (27).

Various studies have analyzed the correlation between the spread of COVID-19 and environmental conditions such as temperature, humidity and wind velocity (28–38). The issue of co-infection with other viruses, such as influenza and rhinovirus affecting the respiratory system, has been the subject of several clinical studies as well (39–46). These better known viruses are also seasonal, raising again the issue of the dependence on the environment (29–32, 47–52). There is a growing consensus that environmental factors can have an effect on SARS-CoV-2 via four main mechanisms (53): (a) exacerbating other respiratory conditions, (b) influencing host susceptibility response to infection through immune response modification, (c) modifying viral metabolism and spreading, and (d) altering human behavioral patterns.

The conventional interpretation of the experimental evidence is that, with decreasing temperatures, people tend to spend more time indoors, in poorly ventilated locations, conditions well known for being conducive to increased transmission of viruses. Even if lower temperatures are correlated with the morbidity of SARS-CoV-2 and the mechanism of the effect is plausible, there are still some aspects that require further clarification (46–49).

**Figure 1:**
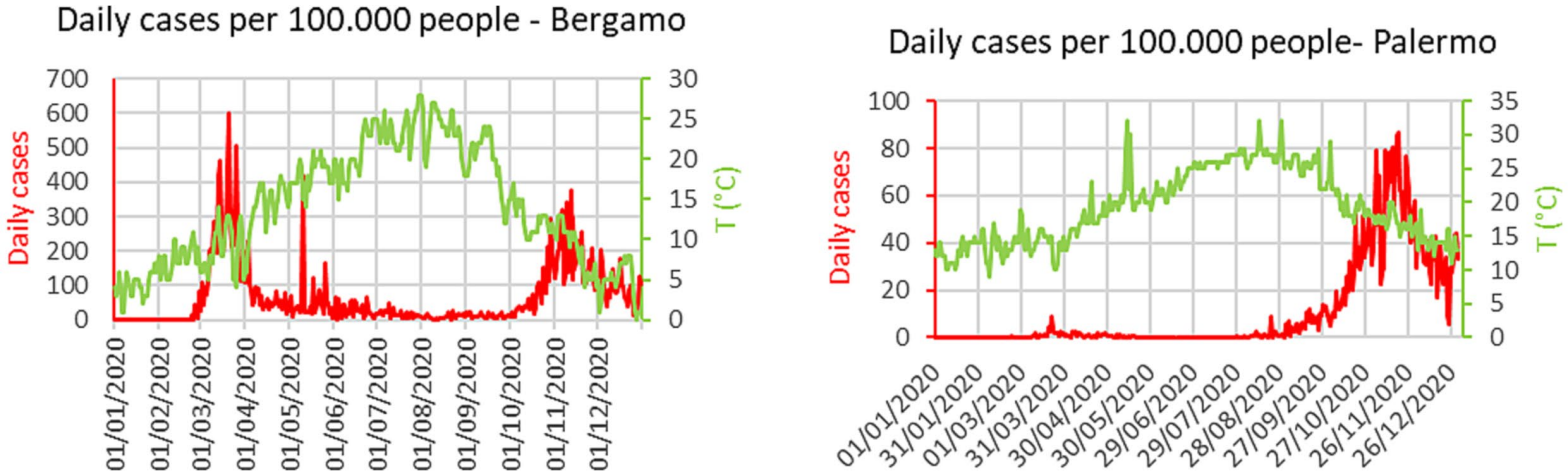
Time evolution of temperature and number of COVID-19 cases. Left: city of Bergamo Italy. Right: city of Palermo Italy.

**Figure 2:**
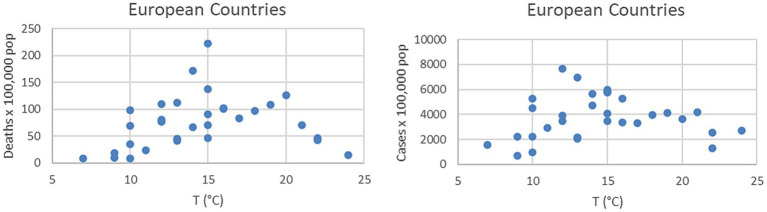
Number of COVID-19 deaths (right) and cases (left) in various European countries vs. the average annual temperature of their capital city. All the details about the values reported in the plots can be found in Appendix A.

The nature of the difficulties of an oversimplified interpretation of the temperature impact can be understood by simple inspection of the plots in Figure 1. In the case of Bergamo, the first major city to be severely struck by the pandemic in Italy, there are peaks of the infection even during periods of raising temperatures. An additional indication, that other environmental factors can play a significant role, is the difference in the absolute values of the temperature, when the outbursts of infections began in the last quarter of the year. In Bergamo, the temperature dropped below 15°C before the number of infections started to rise significantly, whereas in Palermo, there were already signs of the beginning of the outburst at 25°C. A confirmation of the insufficient role of the temperature, to explain the evolution of the COVID-19 pandemic (36, 37), can also be derived from the inspection of data at the European scale. The plots of Figure 2 show the number of infections and excess deaths in 2020 in various European countries versus the average annual temperature. The names of the countries and all the numerical values shown in Figure 2 are reported in Appendix A. At the aggregate level, there is no evidence of correlation between the temperature and the number of infections or deaths attributed to SARS-CoV-2. Indeed, on the continent, some of the countries less affected by the virus are the northern, coldest ones such as Norway and Finland (see Appendix A). Of course, the average annual temperature is a quite coarse indicator and different behavioral and policy factors have certainly played a role in the scatter of the results (54, 55). However, it remains an important observation that some of the coldest countries in the European Union have been affected much less by the pandemic than much warmer ones.

The analysis of the evolution of the new cases at high time resolution emphasizes the problematic nature of the naïve interpretation that the temperature decrease is the only direct cause of the increased spreading of the contagion (36, 37). Indeed, from the plots of Figure 1 it is already evident that the spectral components of the temperature and the evolution of the infections are significantly different. The cases of COVID-19 fluctuate at much higher frequencies than the variations in the ambient temperature (see Section 4).

All the aforementioned pieces of evidence seem to indicate that, even if the temperature certainly plays a crucial role in facilitating the spread of the infection, other environmental factors could also be important, motivating an observational investigation of other potential causes, mainly wind and air quality (pollutants). The objective of the present work consists of substantiating the position that other environmental quantities, and not only the temperature, can have an influence on the morbidity of SARS-CoV-2. The main specificity of the study is the analysis of the weather variations impact on the contagion, mainly through their effect on the air quality and including high frequency components. This is a subject not granted a lot of attention in the literature. Indeed, for example even in the excellent overview reported in (53) a direct link between weather and the level of pollution is not considered, because the study is concerned only with the consequences of long term exposure to poor air quality. The proposed type of investigation requires deploying more sophisticated data analysis techniques than the ones normally reported even recently (56–59).

The lack of fully adequate and well-understood analysis tools has already been identified as one of the main factors rendering metastudies particularly difficult (60). Reaching solid conclusions becomes very problematic because the results of the reported investigations tend to depend also on the statistical methods deployed. To alleviate this issue and to perform high time resolution investigations, non-parametric indicators based on ranking and recurrence analysis in phase space have proved to be particularly appropriate (see Subsection 3.2). In Italy, the relevant information can be obtained with daily frequency, a fact that allows investigating the effects of the weather fast variations on the spread of the contagion.

Italy was one of the first and hardest-struck countries in Western Europe in 2020. During this year, COVID-19 reached very early pandemic proportions, causing 74.000 estimated deaths in 2020 (and about two million infected). A reasonable amount of data is therefore available before the rollout of the first vaccines, at the beginning of 2021. Indeed the objective of the present study consists of investigating the effects of the environmental conditions on the virulence of SARS-CoV-2 and human interventions play the role of confounding factors in this perspective. This is indeed the main reason for limiting the investigation to 2020. Data are available also for 2021 and 2022 but their analysis becomes prohibitively difficult, because of the increased variety and intensity of countermeasures put in place by the government and the healthcare systems.

Another positive aspect of investigating Italian data, in addition to the amount of information collected before the first vaccine rollout, is that its orology is quite varied and therefore the country presents a wide range of climate conditions. The heterogeneous character of Italian geography and industrialization results also in different levels of pollution, allowing the analysis over a quite ample spectrum of air quality conditions. It should also be mentioned that Italy is expected to be quite representative of many other European countries located around the Mediterranean basin.

**Figure 3:**
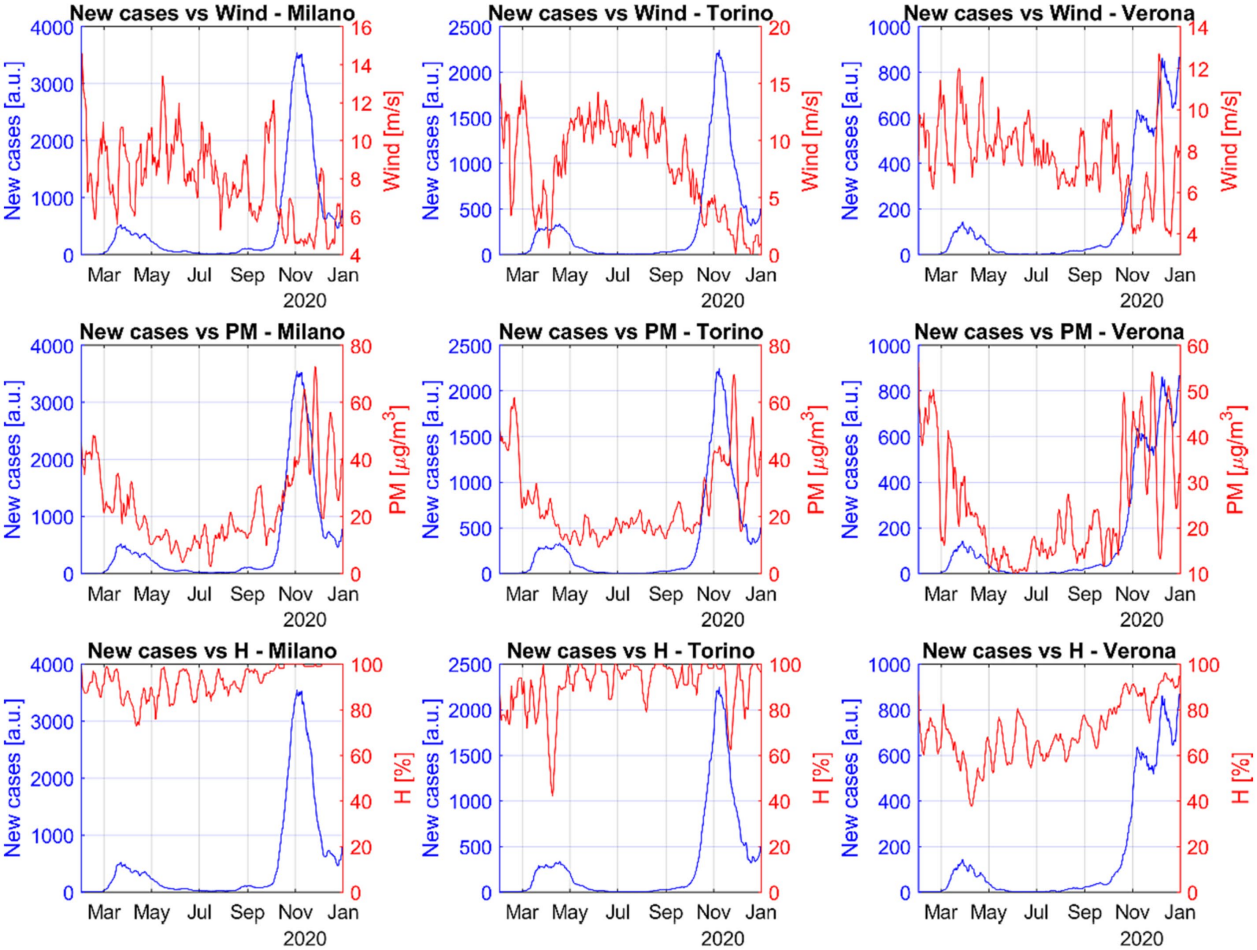
Trend of the number of COVID-19 cases, wind intensity, pollutant amounts and humidity with time for three representative Italian cities. When the winds decrease and the particulates (PM2.5 and PM10) increase, the virulence of SARS-CoV-2 becomes clearly worse. Relative humidity shows a completely different type of correlation.

The main available quantities to investigate the influence of the air conditions on the spread of diseases are the wind intensity, humidity and air pollutants (56, 58). Figure 3 shows, again for representative cases of major Italian urban areas, that also the wind intensity seems to have a strong influence on the number of new cases. This is probably mediated by the pollutants, whose concentration is affected by the air mobility (33, 58–61). Indeed, the number of infections increases significantly when the wind intensity is lower and the concentration of pollutants higher.

Another environmental quantity, investigated in the literature as a potential candidate to explain the effects of the weather on the evolution of the contagion, is relative humidity (RH) (36–38, 62, 63). RH is defined as the ratio of the amount of water vapor present in the air to the greatest amount possible at the same temperature: $RH=p_{H_{2}O}/p_{H_{2}O}^{*}$ where $p_{H_{2}O}$ is the partial pressure of water vapor and $p_{H_{2}O}^{*}$ is the equilibrium vapor pressure of water. As will be shown in detail in Sections 4 and 5, RH has a positive correlation with the number of cases of a strength comparable to the negative one of the temperature. However, RH is highly correlated with temperature, making it very difficult to disentangle the relative influence of these two variables. This is another aspect that needs advanced analysis techniques to be clarified.

The paper is organized in a quite traditional way. The next section provides a brief description of the main SARS-CoV-2 characteristics and overviews the literature on the relationship between the spread of the disease and environmental conditions. Section 3 is devoted to materials and methods: it describes in detail the database of a representative set of major Italian urban areas, the data pre-processing implemented and the statistical tools deployed for the analysis. The main results, obtained by the application of non-parametric indicators based on ranking, are the subject of Section 4. In Section 5, the outputs of the recurrence analysis in phase space are reported, to provide a completely independent view of the matter. The last section is devoted to the summary, discussion, and conclusions.

## 2. SARS-CoV-2 transmission and environmental conditions

In this section, to render the paper self-contained, a brief description of SARS-CoV-2 main characteristics is provided. An overview of the basic literature on the relationship between SARS-CoV-2 transmission and environmental conditions is also reported.

### 2.1. SARS-CoV-2

The SARS-CoV-2 is an enveloped, positive-sense, single–stranded RNA virus causative agent of COVID-19 (64). The associated fatalities are believed to be due to hypercytokinemia, a severe reaction of the immunity systems, characterized by an uncontrolled release of pro-inflammatory cytokines (65). Even if the pathogenesis of SARS-CoV-2 deadly infections is not completely clear, the life cycle of the virus in the human lungs is known to consist of several phases, ending with SARS-CoV-2 reaching the epithelial alveoli, trachea and bronchial tract and replicating in these cells. The main entry door of the pathogen is the angiontensin converting enzyme 2 (ACE2).

The SARS-CoV-2 is aerosolized through talking or exhalation, explaining its virulence and quick spreading (6–8). Indeed bioaerosols are ubiquitous and can be found both indoors and outdoors. The main routes of SARS-CoV-2 infection are considered to be human-to-human transmission via respiratory droplets and contact with contaminated surfaces. More recently evidence has emerged that outdoor viral diffusion *via* aerosols is also a possible pathway (6–8).

### 2.2. Weather conditions

The mechanisms of influenza diffusion have been investigated and debated for more than half a century. The basic modes of transmission are known to be direct contact, indirect contact, droplet transmission, and airborne transmission. The details of the various routes are not fully understood but it is believed that viruses of the influenza type are mainly transmitted through close contact. The effects of the weather on the spreading of these viruses are less known. No laboratory animal displays exactly the symptoms of humans and therefore animal studies are difficult to extrapolate to the natural transmission in our species. Epidemiological and observational human studies are also very difficult to interpret due to the presence of confounding factors and the lack of sufficiently complete databases (39–46).

The influence of weather conditions on the morbidity of SARS-CoV-2 has also been analyzed recently. The main variables considered are temperature, humidity, wind and enthalpy (56–58). In addition to the evidence that temperature regulates the survival of SARS and MERS, these studies are motivated by the evidence of strong seasonality in the spread of the epidemic (33, 59–61). Given the potential role of various confounding factors linked to geography and population size, the available analyses have provided contradictory results. Some studies have revealed a quite strong correlation with temperature, whereas others show a very small correlation or even a negative one (66, 67). The same uncertainties are true for humidity and wind, even if these factors seem to have a lower incidence. In the attempt to resolve these issues, quite comprehensive meta-analytic studies have been performed, which seem to indicate that the influence of the temperature is significant, whereas humidity and wind roles in the spread of the disease are less clear (67, 68). The possible combined effects of these factors have also been analyzed. In particular, various studies on the influence of enthalpy have been published (69).

Notwithstanding the aforementioned uncertainties, the seasonality of viral transmission has been mainly linked to the temperature, since people tend to dwell more inside in colder weather; closer contact and poorly ventilated buildings are obvious conditions increasing the virulence of microorganisms, characterized by airborne transmission (70). Cyclic resistance of the host to infection has also been attributed to seasonal fluctuations in melatonin (71). Vitamin D deficiency, which is linked to the seasons as well, has been invoked as a possible cause (72). Viral stability has also been linked to UV radiation, in agreement with previous evidence that single-stranded RNA viruses can be inactivated by UV radiation (73–75).

The effect of humidity on viral diseases has not been completely clarified. However, there is a consensus that viruses with a lipid envelope, such as those of influenza, are more stable at a lower RH. Other studies showed that non-lipid enveloped viruses of the rhinoviruses and adenoviruses survive longer at high RH (76). Unfortunately, there is not even a clear definition of the line of demarcation between high and low RH (68). In any case, it is believed that there are three mechanisms, which could realistically account for the evidence that humidity influences transmission. The first relates to the host defences. Breathing dry air can cause desiccation of the nasal mucosa, affecting mucociliary clearance, which is an important protection for clearing the lungs of particulate matter (77). The second mechanism is at the level of the viruses themselves. As mentioned, there is evidence that humidity affects the stability of influenza virions. The third category of candidate effects is more physic-chemical in nature. The size of the exhaled bio aerosols is larger at high RH. Larger droplets tend to remain airborne for shorter periods and distances, affecting their potential for contamination (78). In reality, all these three types of mechanisms are expected to be simultaneously involved in determining the actual rate of aerosol transmission.

### 2.3. Particulate matter

Air pollution due to particulate matter (PM) is quite complex, originating from both anthropogenic sources, such as power generation and traffic, and natural phenomena, such as dust and biomass combustion. In urban areas, PM concentration is due to particles of different sizes: ultrafine particles PM0.1 with diameter < 0.1 μm, fine particles PM2.5 with diameter < 0.25 μm and coarse particles PM10 with 0.25 μm < diameter < 10 μm. Inhalable fine and coarse particles can contain not only chemicals and salts but also biological species, such as protein and lipids, and have been associated with increased morbidity and mortality.

Coarse particulates deposit mainly in the upper airways. Fine particulates can reach the lower respiratory tract while ultrafine PM are deposited in both the upper and lower respiratory tracts. PM2.5 particulate consists of an inert carbonaceous core, which is covered by sulphate, nitrate, organic chemicals, and metals. On these structures, additional organic pollutants, such as bacteria and viruses can easily be adsorbed. Epidemiologic surveys have indicated that high levels of PM2.5 can have adverse health effects because they can be deposited quite deeply into the lungs. Together with PM10, fine particulate once inhaled can cause inflammation, oxidative and DNA damage, triggering various cardiovascular, pulmonary, and nervous systems diseases (65).

In synthesis, the available evidence suggests that air pollution may increase SARS-CoV-2 risk of infection and COVID/19 associated mortality though two main paths: (a) by modifying the host susceptibility to infection and capability of reaction, and (b) by elevating the incidence of comorbidities (66). In particular, exposure to particulate matter can influence the upregulation of proteins (ACE2 and transmembrane protease serine type 2) necessary for viral entry, leading to higher viral load and therefore elevating the risk of severe COVID-19. More details can be found in (66) but basically all the reported studies are concerned with the effects of long term exposure to poor air quality conditions. To the authors’ knowledge, the effects of the fast weather variations on SARS-CoV-2 infectivity, using advanced data analysis tools, have never been investigated and reported in the literature.

## 3. Data sources, data pre-processing, and main statistical tools

The present section is divided into two subsections, the first of which is an overview of the database built and the sources of the information it contains. A description of the pre-processing, required to convert the inputs to a format suitable for the following investigations, is also covered in detail. The subject of Subsection 3.2 is the description of the analysis tools deployed, some based on nonparametric statistics and others on methods originally developed for the study of dynamical systems.

### 3.1. Database and pre-processing

**Figure 4:**
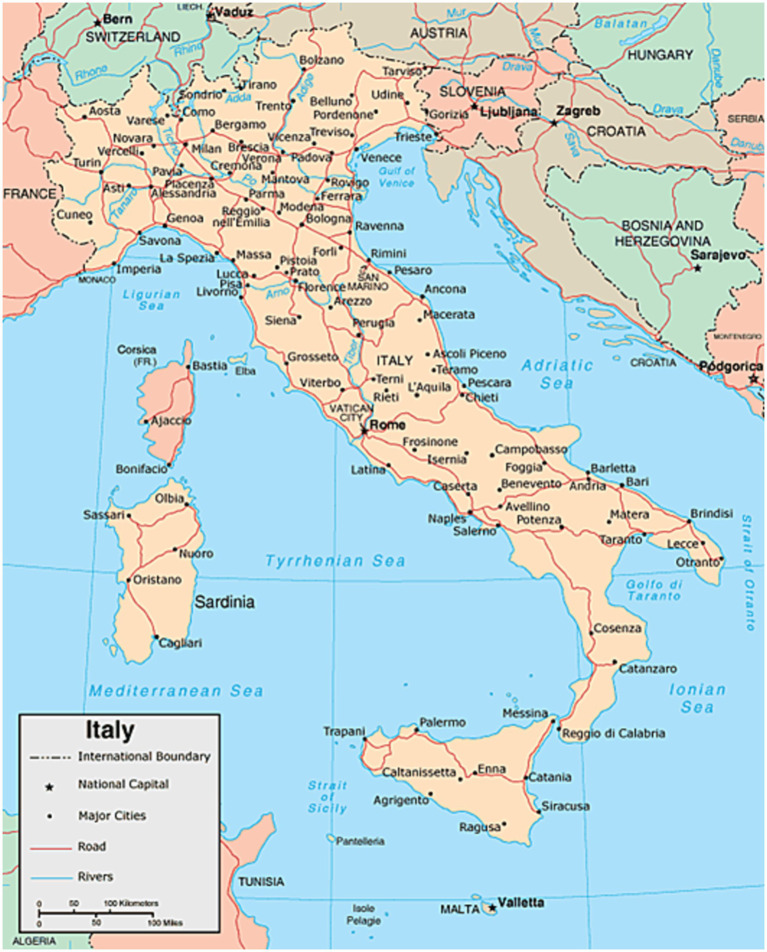
Geographical localization of the cities included in the investigated database.

A specific database (DB) of nine major Italian urban areas has been built. It comprises the following cities: Bergamo, Brescia, Ferrara, Milano, Roma, Torino, Varese, Venezia and Verona (see the map in Figure 4 for their localization in the peninsula). They are among the most densely populated cities and span a latitude range from the industrial north to the center. The DB covers the entire year 2020 before the roll-out of any vaccine. The time resolution is daily. The main characteristics of these cities are reported in Table 1.

**Table 1:** Main characteristics of the representative Italian cities chosen for the survey.

Cities	Coordinates	Elevation	Population	Density
BERGAMO	45°41′42″N 9°40′12″E	249 m s.l.m.	120,390	2,997,76 ab./km^2^
BRESCIA	45°32′20″N 10°13′13″E	149 (min. 101 max. 863) m s.l.m.	196,586	2,176,07 ab./km
FERRARA	44°50′07.07″N 11°37′11.51″E	9 m s.l.m.	130,833	322,92 ab./km^2^
MILANO	45°28′01″N 9°11′24″E	120 m s.l.m.	1,373,517	7,560,51 ab./km^2^
ROMA	41°53′35″N 12°28′58″E	21 m s.l.m.	2,758,839	2,143,02 ab./km^2^
TORINO	45°04′N 7°42′E	239 m s.l.m.	845,606	6,504,16 ab./km^2^
VARESE	45°49′N 8°50′E	382 m s.l.m.	79,639	1,452,21 ab./km^2^
VENEZIA	45°26′23″N 12°19′55″E	2,56 m s.l.m	253,777	610,19 ab./km^2^
VERONA	45°26′19″N 10°59′34″E	59 m s.l.m.	257,264	1,293,3 ab./km

The data about the number of infections has been provided by the European Centre for Disease Control (78). The environmental data have been collected by the Servizio Meteorologico dell’Aeronautica Militare and consist of the following entries: temperature (in degrees C), wind intensity (in Km/h), and relative humidity (in %). The particulates PM10 and PM2.5 (in μg per cubic meter) have been derived from the websites of the Agenzia Regionale Protezione Ambientale. The two Italian agencies sources of the data are national institutions, which provide information already fully validated. The choice of both the urban areas and the independent variables has been motivated by the need to have consistent data of acceptable quality (meaning without missing entries, without changes of standards or definitions etc.) for the entire year 2020.

For the purposes of the present analysis, some form of pre-processing is necessary, even if the data are provided by the most important European, national and regional agencies. The first form of pre-elaboration consists of applying a seven-days moving average to all the time series. This is motivated by the spurious features of the data collection for the number of new infections. Indeed, in all the considered urban areas, the data were not collected and made public uniformly during weekends. Consequently, all the Fourier transforms of the number of infections show a clear weekly frequency component, which is to be considered spurious and is eliminated by the averaging.

The aforementioned averaging alleviates another difficulty, the delay between the moment of the infection and the first symptoms being officially detected. Again a weekly average is expected to remedy also this problem. To corroborate this fact, it has been checked that the analysis tools deployed (see next Subsection) are constant over a period of 7 days or slightly longer.

Another important form of data treatment relates to the quantification of the particulate matter. Indeed, the values of PM2.5 and PM10 have been averaged because they are not always consistently available. This probably causes a slight underestimate of the influx of these two variables on the number of infections.

### 3.2. Analysis tools

To investigate the mutual influence between the number of cases and the environmental variables, two important characteristics of the problem have to be kept in mind: first, the phenomena involved are strongly nonlinear and secondly the data are quite sparse. Consequently, simple indicators, such as the Pearson correlation coefficient, are of limited use because they can pick up only linear effects. Multivariate regression is also out of the question because it does not provide stable results with the limited entries available. Small variations in the data, at the level of the uncertainties, typically change completely the outputs of even the most sophisticate routines. The fact that the number of entries in the database is limited prevents also the use of information theoretic tools, such as mutual information or transfer entropy, which require the calculation of the probability density functions of the variables involved.

To handle the aforementioned difficulties two main types of data analysis techniques have been deployed: statistical indicators and dynamic system analysis. Nonparametric indicators based on ranking belong to the first class: among these, the two most widespread and effective are the Spearman’s and Kendall’s rank correlation coefficients (79). Recurrence plots and derived quantities are the most suitable tools of the second category (80, 81).

#### 3.2.1. Nonparametric statistical indicators based on ranking

The Spearman’s correlation coefficient or Spearman’s *ρ* is a nonparametric measure of the dependence between the rankings of two variables (79). While Pearson’s correlation reflects only the linear relationships between quantities, Spearman’s *ρ* quantifies how well the relationship between two variables can be represented by a monotonic function, without any linearity assumption. In other words, Spearman’s correlation quantifies the monotonic relationships whether they are linear or not. Therefore, the Spearman correlation between two variables is high when observations have a similar rank, i.e., the relative position of the observations within the variables is similar between the two variables. On the contrary, it is low when samples of the two variables have a dissimilar ranking. Consequently, in the case of non-repetitions in the data, the Spearman’s correlation coefficient assumes the values +1 or − 1 when the two variables are a perfect monotone function of each other.

In more detail, given a sample of *n* observations (*x_1_,y_1_*),….,(*x_n_,y_n_*)of the joint random variables *X* and *Y*, the values are ranked first to *R(X)* and *R(Y)*. Then the Spearman’s *ρ* can be calculated as:


(1) $$\rho =\frac{\mathrm{cov}\;\left[R\left(X\right),R\left(Y\right)\right]\;}{\sigma_{R\left(Y\right)}\sigma_{R\left(Y\right)}}$$

where Cov indicates the covariance between two variables.

In its turn, the Kendall’s correlation coefficient or Kendall’s τ is a statistical indicator meant to measure the ordinal association between two variables, based on the similarity of the data orderings between the quantities (79). In other words, the Kendall’s τ rank correlation measures the similarity of the orderings of the data when the quantities are ranked. In more detail, again let us indicate with (*x_1_,y_1_*),….,(*x_n_,y_n_*)the observations of the joint random variables *X* and *Y*. For simplicity all the values *x_i_* and *y_i_* are considered unique; this allows neglecting the problem of ties but more sophisticated treatments taking them into account are available. Any pair of observations (*x_i_,y_i_*) and (*x_j_,y_j_*) are called concordant if either both *x_i_ > x_j_* and *y_i_ > y_j_* are verified or both *x_i_ < x_j_* and *y_i_ < y_j_* are verified. If neither of these couples of conditions holds, the pairs of observations are said to be discordant. With this nomenclature the Kendall’s correlation coefficient is defined as:


(2) $$\tau =\;\frac{\left(\begin{array}{l} \mathrm{number}\;\mathrm{of}\;\\ \mathrm{concordant}\;\mathrm{pair} \end{array}\right)-\;\left(\begin{array}{l} \mathrm{number}\;\mathrm{of}\\ \mathrm{disco}r\mathrm{dant}\;\mathrm{pair} \end{array}\right)}{\left(\begin{array}{c} n\\ 2 \end{array}\right)}$$

where *n* is the number of data points. The Kendall’s correlation coefficient ranges between −1 and + 1.

Intuitively, if the agreement between the rankings of the two quantities is perfect (the disagreement is perfect), the Kendall’s τ has value 1 (the coefficient has value −1). For two independent variables, the value of the Kendall’s τ approximates zero. An explicit equation to calculate the Kendall’s correlation coefficient is:


(3) $$\tau =\frac{2}{n\left(n-1\right)}\sum_{i<j}\mathrm{sgn}\left(x_{i}-x_{j}\right)\mathrm{sgn}\left(y_{i}-y_{j}\right)$$

To summarize, the Spearman’s and Kendall’s correlation coefficients are purely statistical indicators (79). The Spearman’s ρ quantifies only monotonic dependencies between quantities, whereas the Kendall’s τ is more general.

#### 3.2.2. System dynamics indicators based on recurrence plots

Different indicators, devised to investigate the dynamics of complex systems, can be derived from recurrence analysis (80). Recurrent behavior, ranging from periodicities to irregular cyclicities, is a distinct aspect of most natural processes. Seasonal variations are an example of quite regular phenomena, whereas heart beat intervals can vary more substantially. The recurrence of states in phase space has been recognized since a long time as a fundamental property of deterministic dynamical systems. It is an aspect particularly relevant for the understanding of nonlinear and chaotic dynamics.

One of the most effective tools to investigate recurrent behaviors are the so-called recurrence plots (RPs) (80). An RP is the plot of a matrix, which describes how phase space trajectories visit the same regions in phase space. Mathematically an RP is the visualization of a recurrence matrix:


(4) $$RP_{ij}=\Theta \left(\epsilon -||y_{i}-y_{j}||\right),y_{i,j}\in R^{m},i,j\in 0,N$$

where *Θ* is the Heaviside function, *N* is the number of samples, “*m*” the dimension of the embedded phase space, ||°|| is a norm, ϵ is a suitably chosen threshold and the *i* and *j* subscripts indicate two time points (80). When the distance of phase space values at times *i* and *j* is smaller than the threshold, the Heaviside function assumes the value of one; otherwise it is zero.

To investigate the relation between two variables, the task of the present work, it is possible to extend the concept to the cross recurrence plots (CRPs), which compare the dynamical behavior of two time series embedded in phase space (81):


(5) $$CRP_{ij}^{xy}=\Theta \left(\epsilon -||x_{i}-y_{j}||\right),x_{i,}\;y_{j}\in R^{m},\in R^{n},i,j\in 0,N$$

where *x_i_* and *y_i_* indicate the two time series (the rest of the notation is the same as the one of Equation 4).

Visually, RPs mostly present single dots and lines; the lines can be parallel to the main diagonal (*line of identity*, LOI) or vertical/horizontal. Lines parallel to the main diagonal are called *diagonal lines* and are the most important for the objectives of the present study. Indeed, the diagonal lines represent the phase space trajectory segments running parallel for some time; in CRPs, they therefore indicate the periods of simultaneous recurrence behavior of the two signals considered. Consequently, the diagonal lines provide information about the similarity of the systems’ behavior in phase space. The properties of CRPs include the nonlinear effects between the signals analyzed and therefore these tools are a very good complement to the more traditional Spearman’s and Kendall’s correlation coefficients (81).

When performing a pairwise comparison for a set of time series, the difficulty resides in setting the value of the threshold ϵ . A common value for all pairs may not be a good solution to capture the similarities. Also, there are no general criteria to set different values for each pair while ensuring at the same time a uniform treatment for all pairs. A solution is to use a fixed recurrent rate for all pairs allowing ϵ to adjust for each pair (80). The recurrence rate is defined by the relation:


(6) $$RR^{ij}=\frac{1}{T^{2}}\sum_{t_{1}=1}^{T}\sum_{t_{2}=1}^{T}CRP_{t_{1},t_{2}}^{ij}$$

The properties of RP are quantified in terms of specific indicators by the sub-discipline called recurrence quantification analysis (RQA) (80). In the perspective of the present work, the main advantage RQA is that it can provide concise, quantitative information, including nonlinear effects, even for short and non-stationary data, when other methods either fail or are simply impossible to apply. In this approach, the following RQA indicators have been used:

A. Determinism (DET)

(7) $$DET=\frac{\sum_{l=l_{\text{min}}}^{N}lP\left(l\right)}{\sum_{l=1}^{N}lP\left(l\right)}$$

where l denotes the length of a diagonal line (it means the number of recurrent points in it), $l_{\text{min}}$ is the minimum length considered for the diagonal structure and $P\left(l\right)$ is the histogram of the diagonal line lengths. DET represents the fraction of recurrent points forming diagonal structures and it accounts for the predictability of the system.

B. Average diagonal line length (L)

(8) $$L=\frac{\sum_{l=l_{\text{min}}}^{N}lP\left(l\right)}{\sum_{l=l_{\text{min}}}^{N}P\left(l\right)}$$

L is interpreted as the mean prediction time as it is the average time that two segments of a trajectory are close to each other.

C. Entropy of diagonal lines (ENT)

(9) $$ENT=-\sum_{l=l_{\text{min}}}^{N}p\left(l\right)\log p\left(l\right),\;p\left(l\right)=\frac{P\left(l\right)}{\sum_{l=l_{\text{min}}}^{N}p\left(l\right)}$$

ENT is a complexity measure of the distribution of diagonal lines, with low values for uncorrelated noise and high values for complex dynamic evolution.

## 4. Italy: statistical analysis in the temporal and frequency domain

**Figure 5:**
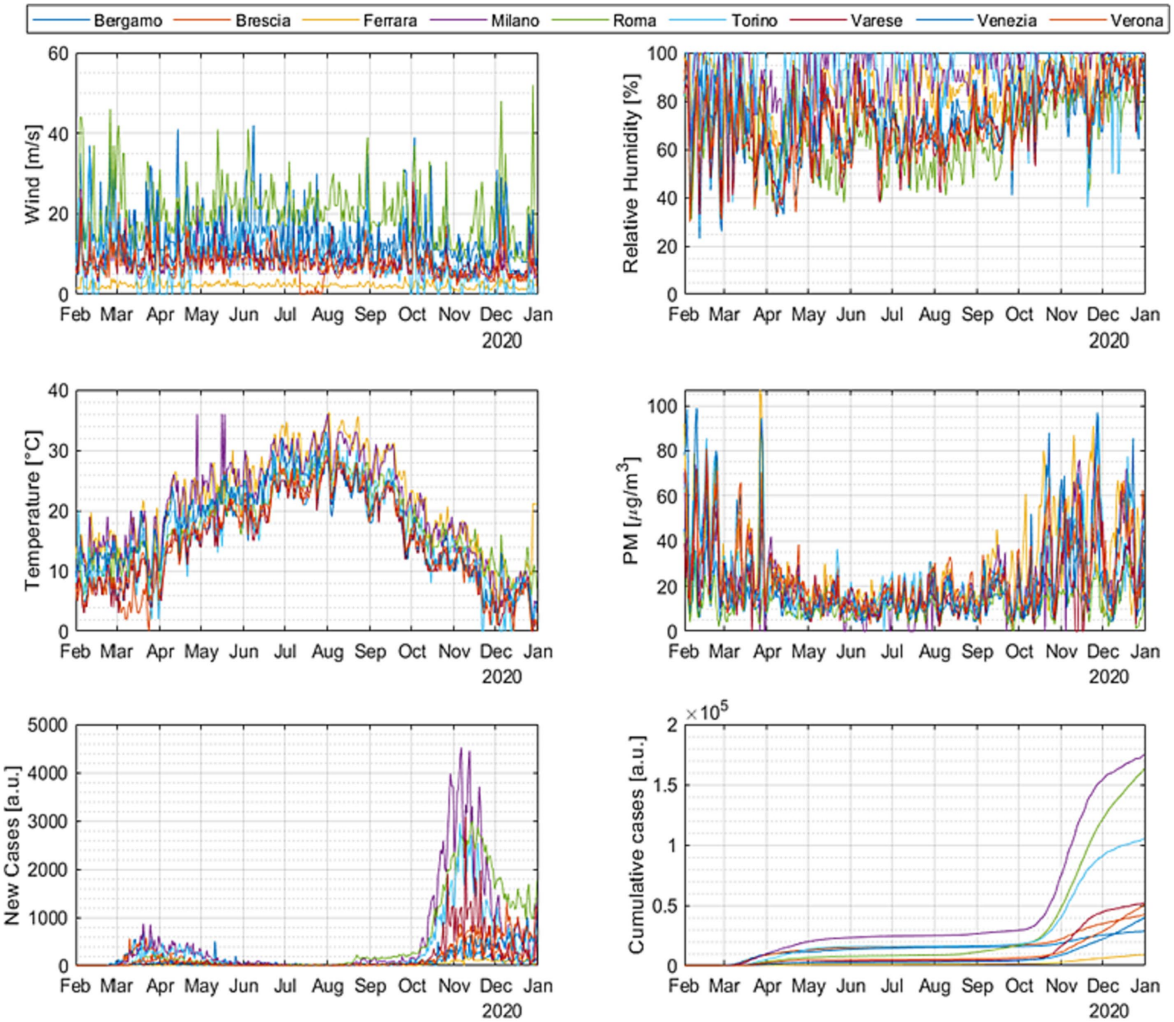
Time evolution of the number of infected people and the various candidate correlates for all the most representative cities investigated.

A systematic analysis of the data described in Subsection 3.1 has been performed. The time evolution of the infections and the potential correlates is shown in Figure 5 for all the cities in the DB. The signals’ evolution in the temporal domain confirms the observations already mentioned about the behavior of the time series. The effect of the temperature is evident and consistent; the decrease of the temperature in winter corresponds to an increase in the number of cases in all cities.

**Figure 6:**
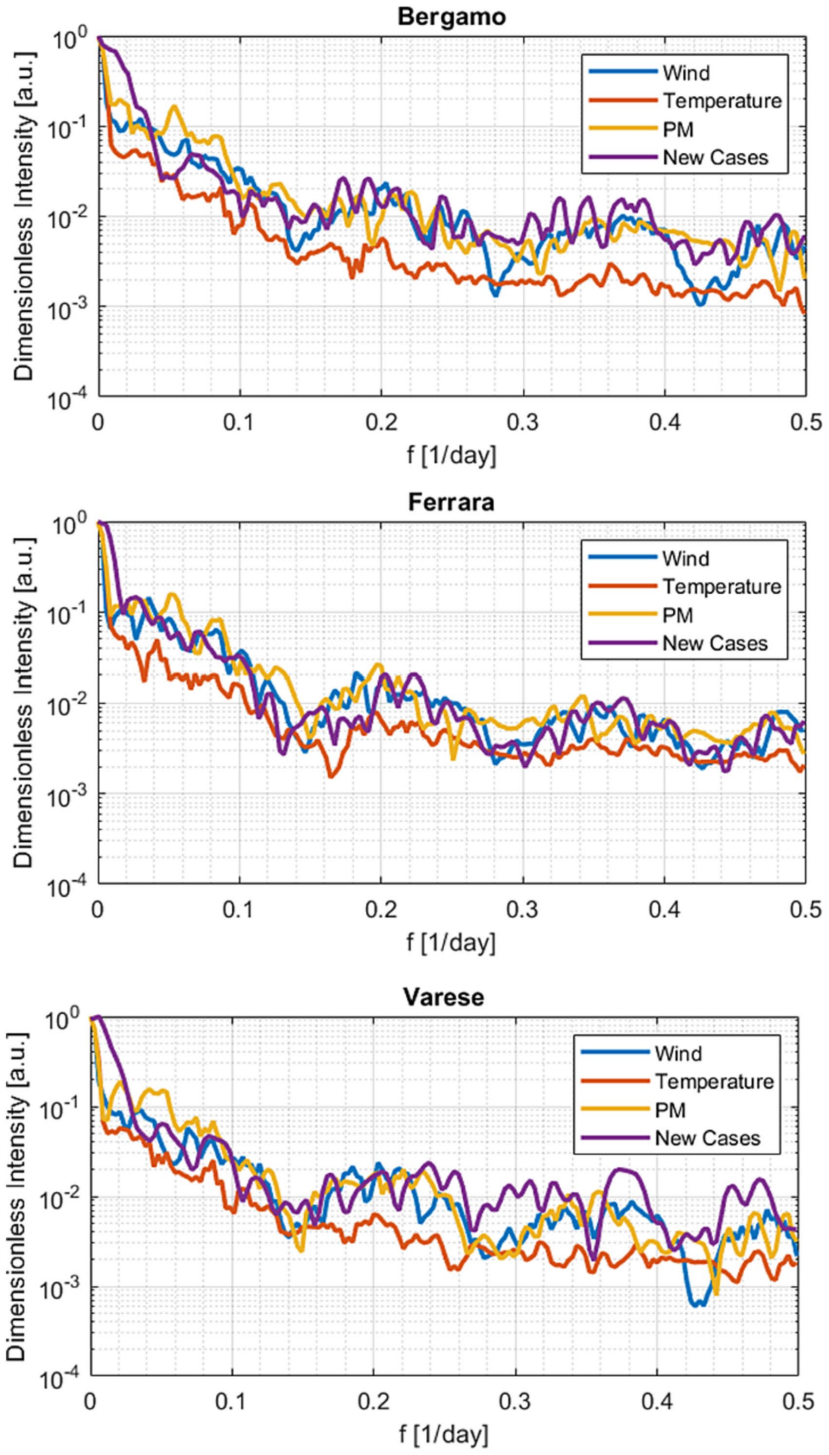
Fast Fourier transform of the quantities in the database for three representative cities.

However, temperature alone cannot account for all the aspects of the infection evolution. A systematic analysis of the trends in all the Italian cities analyzed indicates that the outburst of the COVID-19 cases can occur in phase with the temperature increases, decrease, and even remains stationary. Acceleration of morbidity can take off in different cities at significantly different levels of absolute temperature. Such an insufficient influence of the temperature on the COVID-19 consequences can be appreciated also by considering the fast Fourier transform (FFT) of the signals, reported in Figure 6 for some representative cases (the FFTs for all the cities in the DB are reported in Appendix B). The Fourier analysis reveals that the high frequency variations in wind intensity and pollutants are more similar to the ones of the new infections than those of the temperature in practically all climatic regions investigated. The situation motivates considering the trends of the COVID-19 cases with the evolution of the air quality and wind. On the other hand, simple visual inspection and frequency analysis are not sufficient to understand the involved interconnections between these complex variables. A more advanced statistical analysis is indispensable.

**Figure 7:**
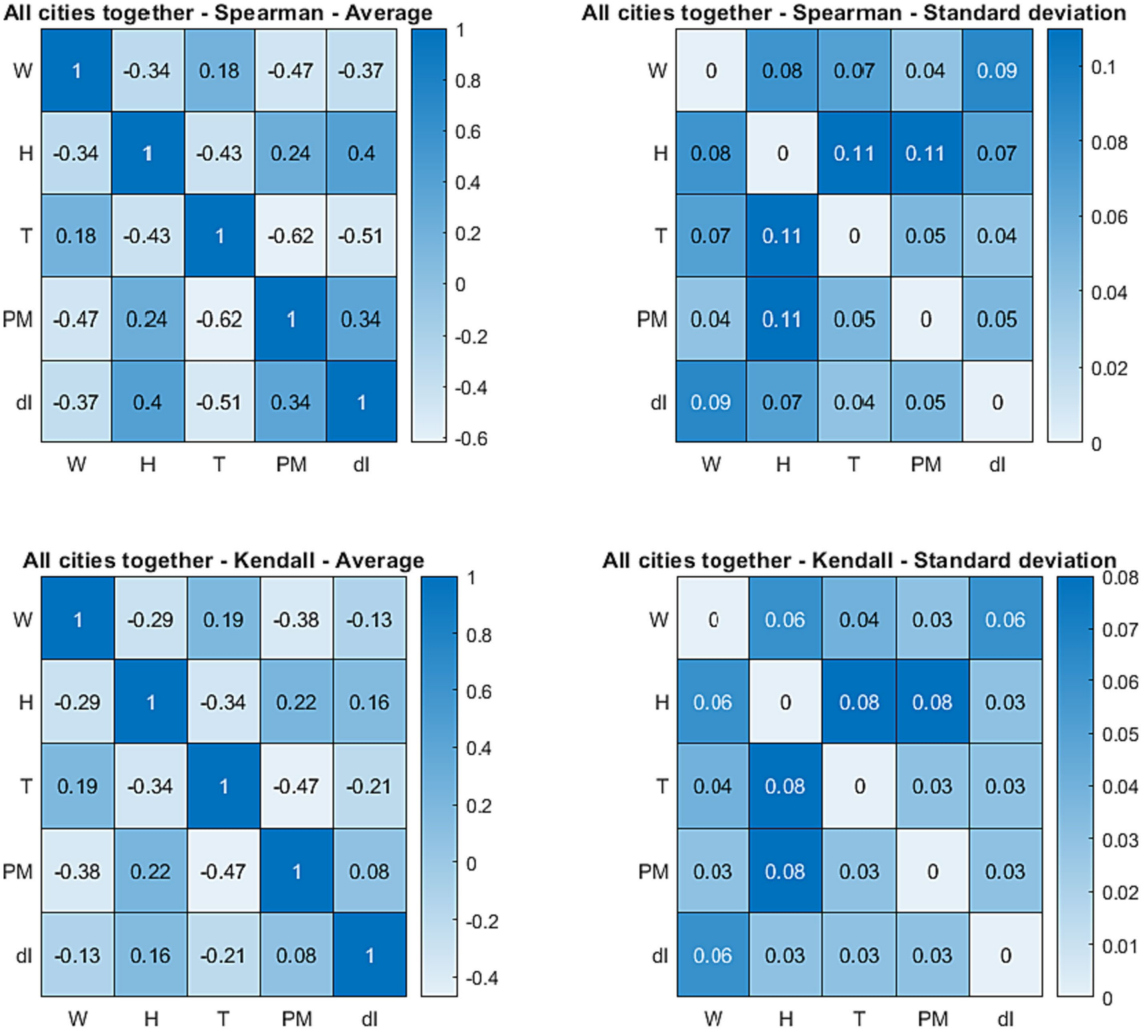
Top row: Spearman’s correlation coefficients mediated over the nine cities (left) and corresponding standard deviations (right) between the quantities in the database. Bottom row: Kendall’s correlation coefficients mediated over the nine cities (left) and corresponding standard deviations (right) between the quantities in the database. W, wind; T, temperature; H, humidity; PM, particulate; dI, number of new infections. In the top row, each cell of the tables reports the Spearman’s correlation coefficient or the standard deviation of the quantities in the corresponding row and column. In the bottom row, each cell of the tables reports the Kendall’s correlation coefficient or the standard deviation of the quantities in the corresponding row and column.

A first global statistical view of their relations can be derived by inspection of Figure 7, in which the average Spearman’s and Kendall’s correlation coefficients, between the main weather and air quality indicators (temperature, wind, humidity, and particulate) and the number of COVID-19 cases, are reported. The standard deviations are also shown in the right column and the corresponding 95% confidence interval can be calculated as usual by multiplying the standard deviation by 1.96 (82). The Spearman’s and Kendall’s correlation coefficients for the individual cities are reported in Appendix C.

Inspection of Spearman’s ρ and Kendall’s τ reveals that the temperature is indeed the variable most correlated with the number of COVID-19 cases; the virus infection is higher (or more prevalent) at lower temperatures. However, humidity, particulate, and wind present correlation coefficients not much inferior to that of the temperature and are typically very similar to each other. It is worth noting that, as shown in the right table of Figure 2, the variances of the correlation coefficients are very low. This means that the obtained correlations are statistically significant. Indeed their confidence levels is above 95% as calculated with traditional statistical tools (82). This conclusion has been confirmed by the analysis of the *p*-values, with the usual null hypothesis assumption of no effect of the various candidate quantities on the number of infections (82).

The most interesting environmental variable is probably the wind, which is not highly correlated with the temperature and has a strong negative correlation coefficient with the number of infections. Higher wind intensities probably tend to reduce the virulence of the contagion by reducing the density of particulate matter (65). Humidity has strong positive correlation coefficient with the number of new cases but it is also highly anticorrelated with temperature. This is most likely due to the effect of precipitations on both humidity and temperature. Unfortunately, there are insufficient data on rainfalls to allow performing a sound statistical analysis for the period investigated in the present work but it is well known that precipitations have the combined effect of increasing humidity and decreasing the temperature. It is therefore reasonable to conclude that in reality the correlation between humidity and infections is a spurious effect, determined by the strong correlation with the temperature (and the strong influence of the temperature on the number of cases). This interpretation of the statistical analysis is corroborated the observation of the fast virus dynamics at the end of summer. A shown in the plots of Figure 3, at the end of August and in September there are often the first signs of an increase in the number of new infections due to an increment in the particulate, when the humidity does not show any consistent increase yet. In any case, it is important to corroborate the previous observations with the alternative approach of the recurrence analysis as described in the next section.

## 5. Italy: dynamical analysis with recurrence plots

The observations and the analysis of the correlation coefficients, presented in the previous section, suggest that other environmental factors, in addition to the temperature, can play a significant role in the spread of the disease. Indeed wind velocity, humidity, and particulate matter have correlation coefficients statistically significant and comparable to that of temperature. The results of the recurrence analysis tend to confirm such a picture. It should be mentioned though that, contrary to the case of the statistical indicators of the previous section, a normalized version of the RQA indicators is not available. Therefore, it is not possible to attribute a specific meaning to their numerical absolute values. Conclusions have to be derived from their relative amplitude and from their standard deviations.

The number of infections and the temperature are the two variables characterized by the higher level of concomitant recurrences. Humidity, particulate, and wind have a lower but very significant level of all the RQA indicators. These trends are consistently reflected by all the indicators and are therefore to be considered solid. An example is shown in Table 2, which reports the average length of the diagonal lines for all the nine cities in the database. This is the most intuitive indicator but the others present an analogous behavior as can be seen from the complementary tables reported in Appendix D.

**Table 2:** Diagonal line lengths from the joint recurrence plots summarizing the recurrences between the main weather indicators and the number of cases for all Italian cities in the DB.

Average						Standard deviation					
	W	H	T	PM	dI		W	H	T	PM	dI
W	0.000	0.702	0.697	0.495	0.766	W	0.000	0.087	0.050	0.037	0.067
H	0.702	0.000	0.741	0.813	0.933	H	0.087	0.000	0.044	0.100	0.054
T	0.697	0.741	0.000	0.801	0.975	T	0.050	0.044	0.000	0.059	0.036
PM	0.495	0.813	0.801	0.000	0.778	PM	0.037	0.100	0.059	0.000	0.051
dI	0.766	0.933	0.975	0.778	0.000	dI	0.067	0.054	0.036	0.051	0.000

The relation between the candidate explanatory variables, emerging from RQA, is also consistent with what is found with the statistical indicators. Wind and pollutants have almost the same influence on the number of cases, strongly indicative of a combined influence. Humidity has a high level of recurrence with the temperature and therefore it is very likely that the latter has by far the larger impact on the contagion.

## 6. Discussion and conclusions

The analysis reported in the present work about many representative Italian cities reveals that the correlation between the outbursts of cases and the temperature, even if statistically very important, is insufficient to explain all the aspects of the contagion fast dynamics. The wind intensity and the presence of pollutants have also a strong correlation and are also strongly correlated with the number of cases on rapid timescales. Humidity seems also to be a relevant concurring cause of the increased virulence but it is very correlated with and presents the same long term trend as the temperature. These conclusions are corroborated by analysis of the absolute values, of the correlations in both the time domain and frequency domain, performed with mathematically independent techniques using statistical and dynamical indicators. The standard deviations of the obtained quantities are very small and therefore the detected influences are to be considered real and not spurious artefacts of the data.

Given the limited quality and quantity of the data, it is not possible to unravel completely the causal relationships between the environmental quantities and the number of infections. However in terms of comparison with previous studies, as far as the relationship between infections, temperature and humidity are concerned, the results of the present analysis are not dissimilar to those obtained in similar studies (67). Indeed relative humidity is strongly correlated with the temperature. Therefore, it is impossible to determine to what extent it is an independent cause of SARS-CoV-2 increased virulence. On the contrary, the effects of the wind and pollutants on SARS-CoV-2 virulence in Italy seem to be significantly clearer than what one would deduce from the most comprehensive meta-analytic study on the subject and most of the references therein (53, 68). Moreover, the analysis techniques deployed can resolve also the rapid evolution of the influence of the wind on air quality and the virulence of the virus.

A possible interpretation of the experimental evidence is emerging. An explanation of the mechanism of the contagion consists of considering the air pollutants an important vehicle for the outdoors spread of the virus. Lower temperatures, tending to induce people to dwell more in poorly ventilated settings, then increase the outdoor transmission in a sort of amplification. Fresher winds can alleviate the problem by dispersing the pollutants and the contaminated aerosols (61). Of course, the details of the transmission mechanisms facilitated by the pollutants have to be further investigated.

If the interpretation just proposed were even remotely valid, it would contrast some serious misconceptions about SARS-CoV-2 seasonality, which have been used by economic interests on question public health measures such as driving reopening or non-intervention (83). On the contrary, the influence of pollutants raises serious questions about the pressure on people to return to their place of work in urban areas even if not necessary. It should indeed be considered that transport is a major contributor to air pollution (84). First, the exhaust of combustion engines produces a lot of particulate among the variables most correlated with the outbreaks of the epidemic. Moreover, urban traffic can have a very deleterious effect not only by increasing the amount of pollutants but also by causing re-suspension and diffusion of particulate (84). Home working should therefore be much more encouraged whenever possible and supply chains management devoted more attention (85).

In terms of generality, it is reasonable to expect that the obtained results should be representative at least of all the other countries of the Mediterranean area if not also of central Europe. Indeed, there is no reason to believe that the situation would be significantly different in other nations with a temperate climate. The public health recommended policies are also expected to be equally valid for all these other countries. However, there is strong evidence of the infection seasonality being different in tropical countries, where outbreaks often peak in wet seasons (86). Such a difference could be linked to pollen inhalation but will require additional studies.

From a data science point of view, the main recommendation would be a better collection of the data (and this applies, even of if to a different degree, to all countries). Since assessing the influence of the environment on the spread of the SARS-CoV-2 is a very complex multi-causal problem, the quality and quantity of the available information are crucial. For example, a more uniform collection of the data over the entire week, including the weekends, would already improve significantly the situation by providing more and cleaner entries. Higher time resolution would allow deploying more advanced data analysis techniques. In this perspective, to disentangle the effects of the main variables determining the air quality, the authors plan to investigate the potential of causality detection techniques already deployed in other fields (87). Improvements in causal inference are then expected to allow achieving significant progress also in the predictive capability of dynamical and regression risk models (88, 89).

## Data availability statement

The data analyzed in this study is subject to the following licenses/restrictions: Data available on request from the authors. Requests to access these datasets should be directed to https://www.meteoam.it/it/disponibilita-dati.

## Author contributions

AM, MG, and RR contributed to conception and design of the study. CG, RR, and GB organized the database. CG and TC performed the statistical analysis. AM and CG wrote the first draft of the manuscript. All authors contributed to manuscript revision, read, and approved the submitted version.
