# Supplementary material for: Effects of environmental conditions on COVID-19 morbidity as an example of multicausality: a multi-city case study in Italy
Source: Front Public Health. 2023 Oct 25;11:1222389. doi: 10.3389/fpubh.2023.1222389 (PMC10642182; doi:10.3389/fpubh.2023.1222389)
Supplement: Supplementary file 1 [file Data_Sheet_1.pdf]

Appendix A.

Table A: Infections and deaths attributed to COVID-19 in various European countries.

COUNTRY	Population	Cases x 100.000 pop	Deaths x 100.000 pop	T mean (°C)
AUSTRIA	8901064	4096	70	15
BELGIUM	11522440	5661	172	14
BULGARIA	6951482	5802	223	15
CROATIA	4058165	5248	100	16
CYPRUS	888005	2700	15	24
CZECH REP	10693939	6983	113	13
DENMARK	5822763	2897	24	11
ESTONIA	1328976	2221	19	9
FINLAND	5525292	668	10	9
FRANCE	67320216	3945	97	18
GERMANY	83166711	2135	42	13
GREECE	10718565	1307	46	22
HUNGARY	9769526	3366	102	16
ICELAND	364134	1580	8	7
IRELAND	4964440	2052	46	13
ITALY	59641488	3614	126	20
LATVIA	1907675	2228	36	10
LIECHTESTEIN	38747	5735	90	15
LITHUANIA	2794090	5296	70	10
LUXEMBOURG	626108	7666	81	12
MALTA	514564	2569	42	22
NETHERLANDS	17407585	4712	67	14
NORWAY	5367580	945	8	10
POLAND	37958138	3485	77	12
PORTUGAL	10295909	4192	70	21
ROMANIA	19328838	3313	83	17
SLOVAKIA	5457873	3446	46	15
SLOVENIA	2095861	6005	138	15
SPAIN	47332614	4138	108	19
SWEDEN	10327589	4480	98	10
UNITED KINGDOM	68059863	3901	110	12

Appendix B: Analysis in the frequency domain

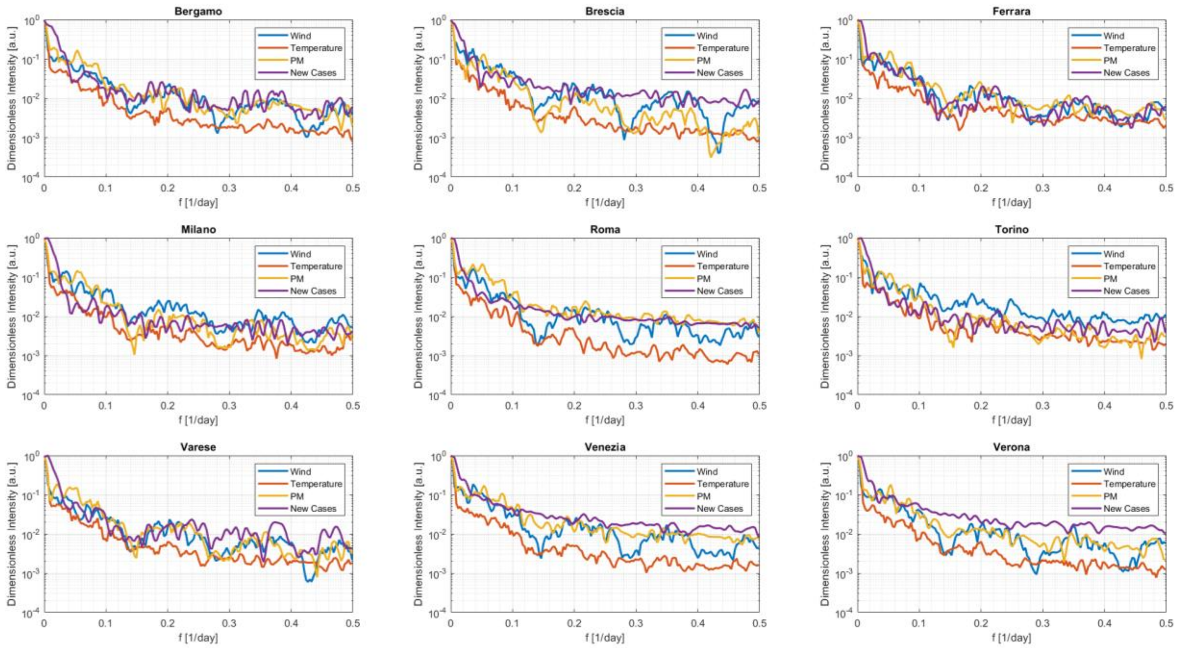

Figure B1: Fast Fourier Transform of the quantities in the database for the cities considered.

Appendix C: Statistical indicators for the individual urban areas.

Figure C1: Spearman's correlation coefficients between the main weather indicators and the number of cases for all Italian cities in the DB.

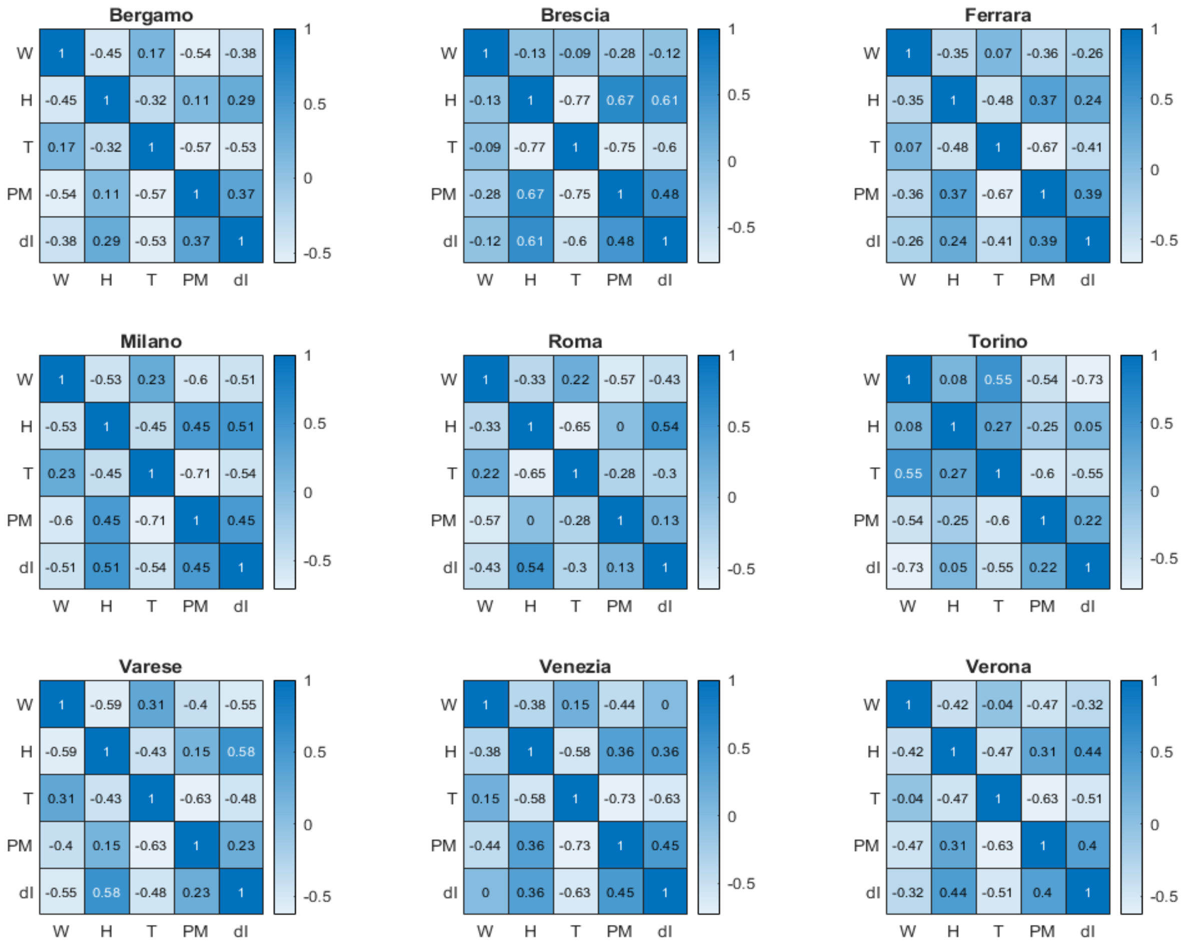

Figure C2: Kendall's correlation coefficients between the main weather indicators and the number of cases for all Italian cities in the DB.

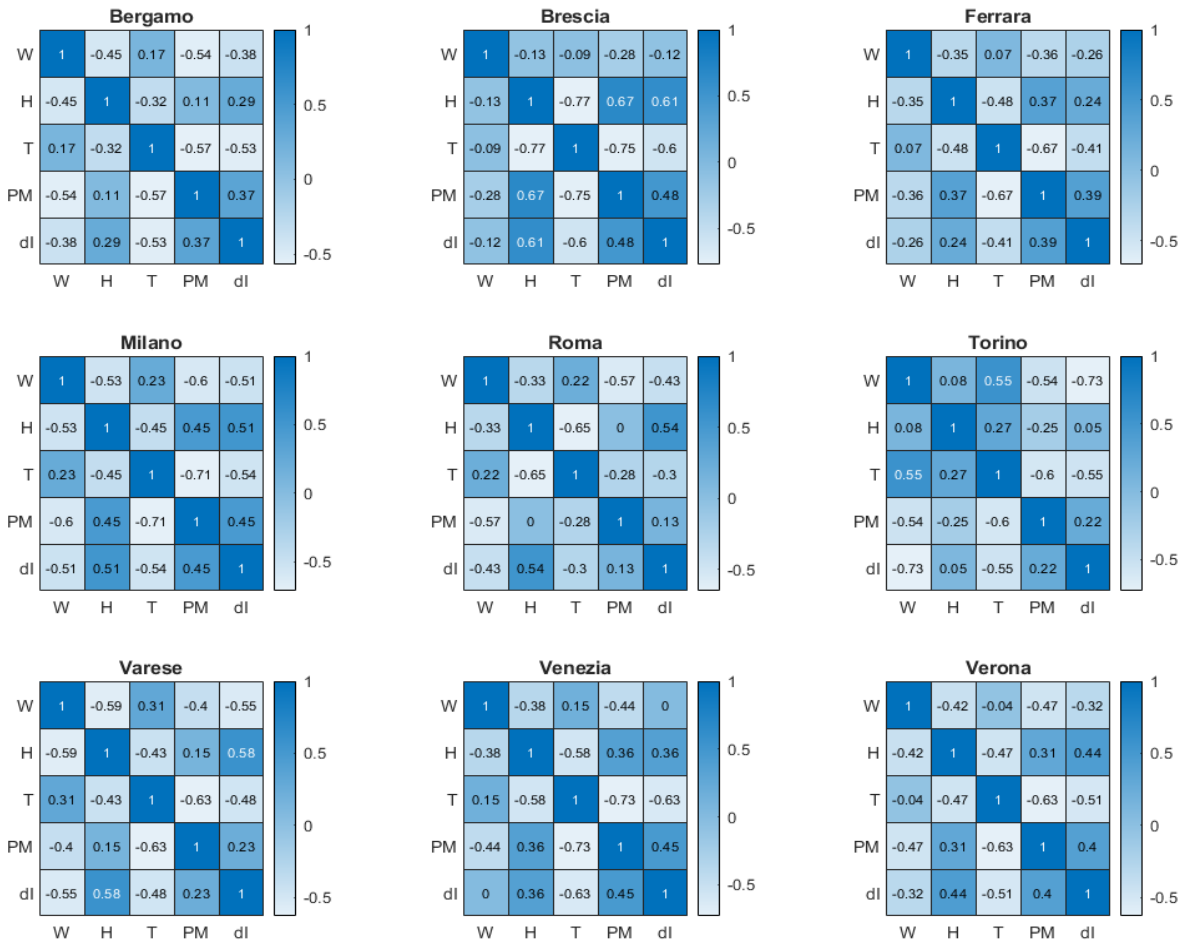

Appendix D1: Recurrence quantification analysis indicators for the entire database.

Table D1: Determinism from the joint recurrence plots, summarising the recurrences between the main weather indicators and the number of cases for all Italian cities in the DB.

Average						Standard deviation					
	W	H	T	PM	dI		W	H	T	PM	dI
W	0.000	0.702	0.697	0.495	0.766	W	0.000	0.087	0.050	0.037	0.067
H	0.702	0.000	0.741	0.813	0.933	H	0.087	0.000	0.044	0.100	0.054
T	0.697	0.741	0.000	0.801	0.975	T	0.050	0.044	0.000	0.059	0.036
PM	0.495	0.813	0.801	0.000	0.778	PM	0.037	0.100	0.059	0.000	0.051
dI	0.766	0.933	0.975	0.778	0.000	dI	0.067	0.054	0.036	0.051	0.000

Table D2: Entropy of the diagonal lengths from the joint recurrence plots, summarising the recurrences between the main weather indicators and the number of cases for all Italian cities in the DB.

Average						Standard deviation					
	W	H	T	PM	dI		W	H	T	PM	dI
W	0.000	0.702	0.697	0.495	0.766	W	0.000	0.087	0.050	0.037	0.067
H	0.702	0.000	0.741	0.813	0.933	H	0.087	0.000	0.044	0.100	0.054
T	0.697	0.741	0.000	0.801	0.975	T	0.050	0.044	0.000	0.059	0.036
PM	0.495	0.813	0.801	0.000	0.778	PM	0.037	0.100	0.059	0.000	0.051
dI	0.766	0.933	0.975	0.778	0.000	dI	0.067	0.054	0.036	0.051	0.000
